# Pharmacokinetics and metabolism of 13-*cis*-retinoic acid (isotretinoin) in children with high-risk neuroblastoma – a study of the United Kingdom Children's Cancer Study Group

**DOI:** 10.1038/sj.bjc.6603554

**Published:** 2007-01-16

**Authors:** G J Veal, M Cole, J Errington, A D J Pearson, A B M Foot, G Whyman, A V Boddy

**Affiliations:** 1 Northern Institute for Cancer Research, University of Newcastle upon Tyne, Newcastle upon Tyne NE2 4HH, UK; 2 Royal Marsden Hospital, Surrey SM2 5PT, UK; 3 Bristol Royal Hospital for Children, Bristol BS2 8BJ, UK; 4 UKCCSG, University of Leicester, Leicester LE1 6TH, UK

**Keywords:** 13-*cis*-retinoic acid, isotretinoin, clinical pharmacology, dosing, drug metabolism

## Abstract

The administration of 13-*cis*-retinoic acid (13-*cis*RA), following myeloablative therapy improves 3-year event-free survival rates in children with high-risk neuroblastoma. This study aimed to determine the degree of inter-patient pharmacokinetic variation and extent of metabolism in children treated with 13-*cis*RA. 13-*cis*-retinoic acid (80 mg m^−2^ b.d.) was administered orally and plasma concentrations of parent drug and metabolites determined on days 1 and 14 of courses 2, 4 and 6 of treatment. Twenty-eight children were studied. The pharmacokinetics of 13-*cis*RA were best described by a modified one-compartment, zero-order absorption model combined with lag time. Mean population pharmacokinetic parameters included an apparent clearance of 15.9 l h^−1^, apparent volume of distribution of 85 l and absorption lag time of 40 min with a large inter-individual variability associated with all parameters (coefficients of variation greater than 50%). Day 1 peak 13-*cis*RA levels and exposure (AUC) were correlated with method of administration (*P*<0.02), with 2.44- and 1.95-fold higher parameter values respectively, when 13-*cis*RA capsules were swallowed as opposed to being opened and the contents mixed with food before administration. Extensive accumulation of 4-oxo-13-*cis*RA occurred during each course of treatment with plasma concentrations (mean±s.d. 4.67±3.17 *μ*M) higher than those of 13-*cis*RA (2.83±1.44 *μ*M) in 16 out of 23 patients on day 14 of course 2. Extensive metabolism to 4-oxo-13-*cis*RA may influence pharmacological activity of 13-*cis*RA.

The retinol derivative 13-cis-retinoic acid (13-*cis*RA) is now an established component of the treatment of high-risk neuroblastoma, despite the fact that early phase II trials conducted with low-dose 13-*cis*RA showed limited clinical benefit in patients with recurrent disease (Reynolds *et al*, 1991; Finklestein *et al*, 1992; Kohler *et al*, 2000). When 13-*cis*RA was administered as a high-dose (160 mg m^−2^ day^−1^), intermittent regimen in a Children's Cancer Group phase III randomised trial, a significant improvement in 3-year event-free survival (EFS) was observed (Matthay *et al*, 1999). Factors that may explain the efficacy observed in the latter study include the higher dose administered and the use of an intermittent dosing regimen, incorporating 2-weeks of 13-*cis*RA followed by a 2 week rest period, on each course of treatment (Matthay and Reynolds, 2000).

Retinoids are susceptible to oxidative metabolism, and the extensive metabolism of all-*trans*-retinoic acid (ATRA) in acute promyelocytic leukaemia (APL) may influence relapse rates (Muindi *et al*, 1992). Analysis of patient samples from a phase I study of 13-*cis*RA previously suggested increasing levels of the metabolite 4-oxo-13-*cis*RA during a course of treatment, although actual concentrations were not quantified (Khan *et al*, 1996).

An additional concern with the administration of 13-*cis*RA is that many neuroblastoma patients are very young. Owing to the large size and number of 13-*cis*RA capsules required to obtain the specified dose, younger children are physically unable to take the drug unless the capsules are opened and the contents mixed with food before administration. This practice raises concerns regarding the actual dose of drug that these patients are receiving, in addition to the possibility that the drug may be unstable during this procedure.

The current study was designed to determine the pharmacokinetics of 13-*cis*RA and the extent of oxidative metabolism when administered to high-risk neuroblastoma patients using an intermittent dosing regimen. In addition, the absorption of 13-*cis*RA when administered following opening of the capsules and mixing the contents with food was investigated.

## MATERIALS AND METHODS

### Patient eligibility and treatment

The study protocol was approved by the UK Trent Multicentre Research Ethics Committee and written informed consent was obtained from patients or parents as appropriate. Patients less than 18 years with a central venous catheter, who were receiving 13-*cis*RA as part of their standard clinical treatment, were eligible.

13-*cis*-retinoic acid (80 mg m^−2^ b.d.) was administered orally as part of a protocol for high-risk neuroblastoma (Matthay *et al*, 1999), starting between 80 and 120 days post-myeloablative and radiation therapy in all cases. Drug was administered for 14 days, with a 14 day break before the next course. Toxicity was assessed by the National Cancer Institute Common Toxicity Criteria (CTC version 2.0) and patients were followed clinically on a 6-monthly basis. In patients who were unable to swallow the capsules, each capsule was snipped with a pair of scissors and the contents extruded into ice-cream or yoghourt before ingestion. Patients were not fasted before administration. On each day of the study, the administration of the studied dose of 13-*cis*RA was performed in hospital, supervised by a trained research nurse and fully documented.

### Patient details

Age and weight together with 13-*cis*RA administration details were recorded for each patient. Glomerular filtration rate (GFR) was estimated according to the equation:

GFR (ml/min)=36.76+1.91^*^weight (kg)−0.47^*^serum creatinine (*μ*mol l^−1^) (Cole *et al*, 2004).

The most recent ALT, bilirubin and creatinine measurements before 13-*cis*RA treatment were obtained from the patients' notes (mean 26 days before administration, maximum 63 days prior).

### Blood sampling and analysis

Blood samples for measurement of concentrations of individual retinoids were obtained from a central line before administration and at 1, 2, 4 and 6 h post-administration. Samples were obtained on days 1 and 14 (first and last day) of treatment courses 2, 4 and 6 following administration of the first dose of 13-*cis*RA on the particular study day. When sampling on these courses was not possible, samples were obtained on days 1 and 14 of an alternative treatment course. Course 1 was not studied in most cases to allow the families and patients to become familiar with the administration processes. Blood samples (5 ml) were collected in heparinised tubes and centrifuged at 1200 g for 10 min at 4°C. Plasma was separated and frozen at −20°C, before analysis using a high-performance liquid chromatography assay, with a limit of quantitation of 0.02*μ*g ml^−1^ for all retinoids. This analytical assay allowed for individual quantification of 13-*cis*RA, 9-*cis*RA and ATRA, in addition to the metabolite 4-oxo-13-*cis*RA, as previously described (Veal *et al*, 2002). All blood and plasma samples were wrapped in aluminium foil to protect them from light, and all sample handling was carried out in dim light. The assay was validated with regard to linearity, reproducibility and stability of the analytes according to standard practice (Shah *et al*, 1992).

### Pharmacokinetics/statistical analysis

A population pharmacokinetic analysis was carried out using 13-*cis*RA plasma concentrations obtained on study day 1 from the first available course of treatment for each of 28 patients (course 1 for five patients, course 2 for 20 patients and courses 3, 4 and 6 for one patient each). Peak and trough 13-*cis*RA levels from days 1 and 14 were determined, together with peak levels of the metabolite 4-oxo-13-*cis*RA on day 1 and 14; these were compared between treatment courses 2, 4 and 6.

Using NONMEM (Beal and Sheiner), a series of population pharmacokinetic models were fitted to 13-*cis*RA data from the first available course of treatment, with all patients included in the analysis. A one-compartment model with first-order absorption (ADVAN2) was fitted using the FOCE estimation method with η, ε interaction. A composite within-subject error model was used together with an exponential between-subject error model for each of the population PK parameters (CL, *V*, *K*_a_). Models were fitted using firstly, a diagonal, and then a full block structure for the between-subject covariance matrix. An absorption time lag was added, which was also allowed to vary across the population. A zero order absorption model with an absorption lag was also fitted (ADVAN1); both the absorption lag time and absorption duration were allowed to vary across the population.

Two further models were fitted to try to more closely model the absorption phase. The first was a ‘transit model’, which assumes that the absorption delay is due to the passage of drug through a chain of transit compartments (Savic *et al*, 2004). The number of transit compartments and the rate at which the drug moves between each of the compartments were estimated from the data. The model was parameterised in terms of CL, *V*, *K*_a_, *N* (the number of transit compartments) and the MTT (the mean transit time to the absorption compartment). The second model was a modified zero-order absorption model with an absorption lag time. This model assumes that the appearance of the drug in a dose compartment is described by a zero-order process over a fixed duration (*D*). Absorption into a central compartment was described by a first-order process with rate parameter *K*_a_. This model was parameterised in terms of CL, V, *K*_a_, LAG and *D*. Both of these models allowed all pharmacokinetic parameters to vary between individuals according to an exponential error model; both were fitted using ADVAN2.

The relationships between pharmacokinetic parameters (apparent clearance, AUC and apparent volume of distribution), peak concentrations of 13-*cis*RA and 4-oxo-13-*cis*RA on days 1 and 14 of treatment, and covariates were examined graphically and either *t*-tests or linear regression applied as appropriate. The pharmacokinetic parameter estimates used were the empirical Bayes estimates obtained from the modified zero-order absorption model, except the peak concentrations. Covariates investigated were age, weight, ALT, bilirubin, creatinine, GFR, sex, method of administration and CTC grade 3/4 toxicity. The logarithm of all pharmacokinetic parameters and continuous covariates was used in preference to the untransformed variables owing to the skewed nature of the data. Empirical Bayes estimates of pharmacokinetic parameters for courses 4 and 6, where data were available, were obtained from the final population model.

The Cox proportional hazards model was used to investigate any relationship between pharmacokinetic end points, including peak levels of 13-*cis*RA and 4-oxo-13-*cis*RA on days 1 and 14 on the first available course of treatment, and time to relapse/progression of disease, relative to the first 13-*cis*RA administration.

## RESULTS

### Patient characteristics and treatment

Twenty-nine children were entered into the study. One patient had to withdraw from the study before samples being taken, owing to removal of their central venous catheter. The study population had a median age of 3.2 years (range 1.1–18.7) and included 16 male and 12 female patients. Patient characteristics for the 28 evaluable patients are given in Table 1.

### Pharmacokinetics

The pharmacokinetics of 13-*cis*RA were best described by a modified one-compartment, zero-order absorption model combined with an absorption lag time (LAG) allowing a full covariance matrix for the random effects. The differential equations for the final model are: 
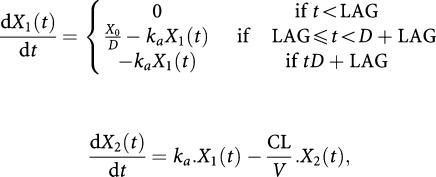
 where *X*_*0*_ is the dose of 13*cis*RA administered at time *t*=*0* and assumed to enter the system at time *t*=LAG; *X*_*1*_*(t)* is the amount of drug in the *dose* compartment at time *t*; *X*_*2*_*(t)* is the amount of drug in the *observation* compartment at time *t*; *D* is the duration of the zero-order input; *k*_a_ is the rate of the first-order absorption; CL is apparent clearance and *V* is apparent volume of distribution.

However, both the transit model and the first-order absorption model with lag time gave very similar parameter estimates and individual fits, despite having slightly larger objective function values (Table 2). Mean population pharmacokinetic parameters were; apparent clearance 15.9l h^−1^, apparent volume of distribution 85 l and absorption lag time 40 min. There was large inter-individual variability associated with these parameters. Clearance had an inter-individual CV of 69%, while both volume of distribution and *K*_a_ had CVs in excess of 100%. Correction of clearance or volume of distribution for body surface area did not reduce the CV (Table 3).

**Figure 1:**
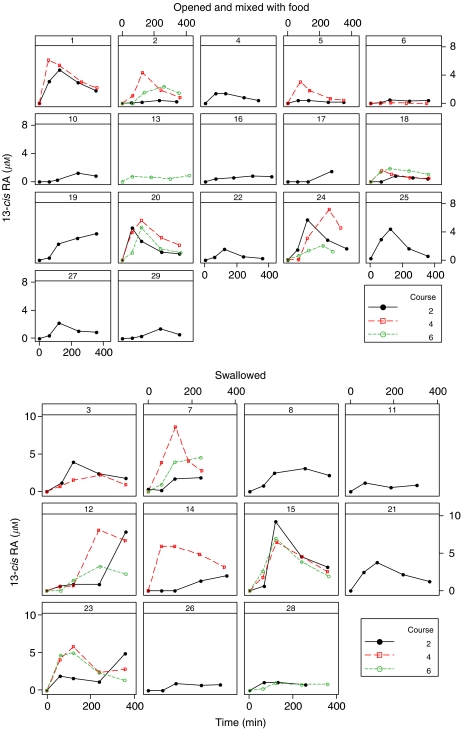
13-*cis*-retinoic acid data; study day 1 for all patients shown by course; separate graphs shown by method of administration. Patient 26 received 13*cis*RA via an NG tube.

**Figure 2:**
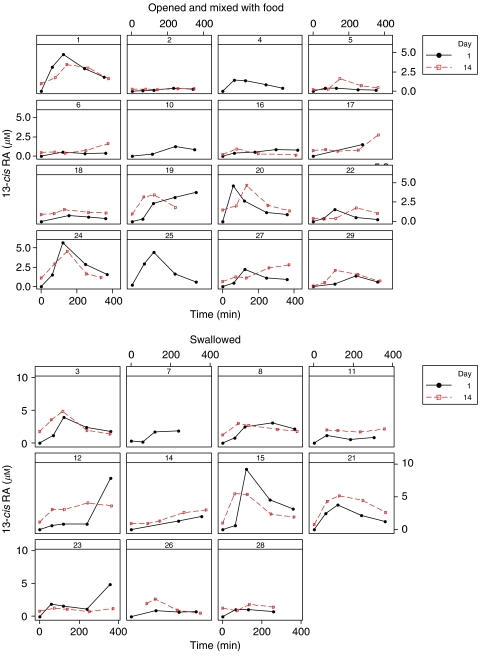
13-*cis*-retinoic acid data; course 2 for all patients shown by study day; separate graphs shown by method of administration. Patient 26 received 13*cis*RA via an NG tube.

There was a large degree of variation between courses in day 1 13-*cis*RA levels (Figure 1; Tables 3 and 4). Whereas some patients showed little variation between courses (e.g. patients 15 and 20), others exhibited large differences (e.g. patients 2 and 7). In contrast, variation in 13-*cis*RA levels between study days (1 and 14) of the same course, in particular course 2, was relatively small (Figure 2A).

**Figure 3:**
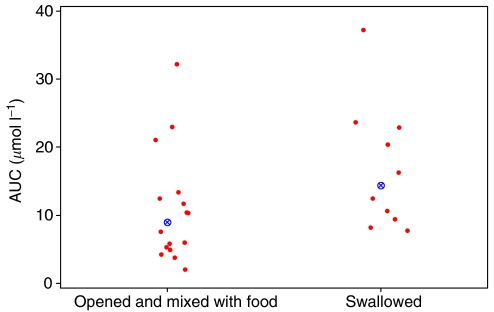
Area under the curve of 13-*cis*RA observed on day 1 of course 2 of treatment in patients who swallowed 13-*cis*RA capsules *vs* those patients for whom the capsules were opened and the contents mixed with food before administration. Median values are identified with a cross.

Owing to difficulties in obtaining stable parameter estimates, the inclusion of covariates into a population pharmacokinetic model was not undertaken. Instead, the relationship between pharmacokinetic parameter estimates and covariates was examined via plots and either *t*-tests or linear regression as appropriate. 13-*cis*-retinoic acid AUC and day 14 peak 4-oxo-13-*cis*RA levels were both found to be related to weight, age and whether or not the capsule had been opened before administration. Higher weight and age were also associated with larger AUCs and peak 4-oxo-13-*cis*RA levels (*P*<0.02 in all cases). Day 1 peak 13-*cis*RA concentrations were found to be linked to method of administration (*P*<0.02). When 13-*cis*RA capsules were swallowed, AUC was found to be on average 1.95-fold larger (95% confidence interval (CI) 1.16, 3.28) than when capsules were opened; day 1 peak 13-*cis*RA levels were 2.44-fold higher (CI 1.23, 4.83) and day 14 peak 4-oxo-13-*cis*RA levels were 2.65-fold higher (CI 1.41, 5.00) when capsules were unopened. Figure 3 shows AUC values for 13-*cis*RA observed on day 1 of course 2 of treatment in patients who swallowed 13-*cis*RA capsules *vs* patients for whom the capsule contents were mixed with food.

Larger absolute doses of 13-*cis*RA were associated with higher day 14 peak 4-oxo-13-*cis*RA levels (*P*=0.01). A larger 13-*cis*RA apparent volume of distribution was associated with higher creatinine levels (*P*=0.02), but none of the covariates were found to be related to apparent clearance of 13-*cis*RA, peak 13-*cis*RA levels from day 14 or peak 4-oxo-13-*cis*RA levels from day 1.

### Oxidative metabolism

**Figure 4:**
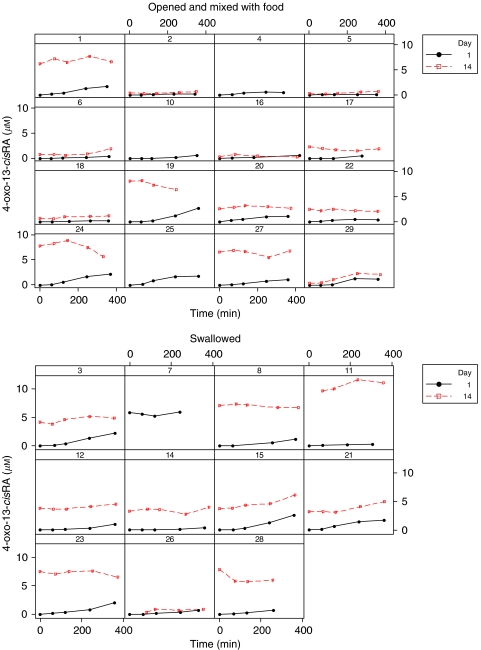
4-oxo-13-*cis*RA data; course 2 for all patients shown by study day; separate graphs shown by method of administration. Patient 26 received 13*cis*RA via and NG tube.

Extensive accumulation of 4-oxo-13-*cis*RA occurred in all patients during each treatment course, with plasma concentrations higher than those of 13-*cis*RA on day 14 of course 2 of treatment in 16 out of 23 patients for whom data were available (Figure 4; Table 4). In course 2, peak concentrations of 4-oxo-13-*cis*RA increased from 0.2 to 5.9*μ*M on day 1 to 0.7–11.6 *μ*M on day 14 of treatment, as compared with 13-*cis*RA plasma concentrations of 0.3–5.5 *μ*M on day 14. Similar increases in the level of metabolite over the 14 days of treatment were observed in subsequent courses (Figure 4). No other retinoic acid metabolites were detected in plasma samples of patients receiving 13-*cis*RA and concentrations of ATRA accounted for less than 5% of total retinoids in all samples.

### Clinical response and toxicity

Of the 28 evaluable patients, 13 (46%) were alive with no disease at follow-up. Time to follow-up ranged from 18 months to 4 years. Of the remaining patients, three (11%) were alive with disease progression and 12 (43%) had died following disease relapse. There was a greater likelihood of relapse for patients with higher day 14 peak 4-oxo-13*cis*RA plasma concentrations (*P*=0.014; Cox regression analysis). No other pharmacokinetic parameters were related to time to relapse or survival.

Treatment was reasonably well tolerated, although several patients had persistent haematological toxicity following the previous myeloablative therapy. Nine patients experienced some form of mild skin toxicity (eight CTC grade 2, one grade 3), with only one report of mild cheilitis. Hypercalcaemia (grade 2 or 3) was reported in two patients. There was no evidence to suggest that any of the toxicities observed were linked to the pharmacokinetics of 13-*cis*RA or its metabolite.

## DISCUSSION

The addition of 13-*cis*RA to the treatment of high-risk neuroblastoma has been shown to improve 3-year event-free survival rates. Nevertheless, despite the addition of 13-*cis*RA to high-dose myeloablative chemotherapy treatment protocols, more than half of patients still suffer relapse within 3 years. While in some patients relapse is mostly influenced by the biology of the tumour, the high degree of variability in the pharmacokinetics and metabolism of 13-*cis*RA observed in this study suggests that there is scope for further improvements based on individualisation of dosing or schedules.

A series of population pharmacokinetic models were fitted to 13-*cis*RA plasma concentration–time data from 28 children with high-risk neuroblastoma. Following detailed analysis of these data, the pharmacokinetics of 13-*cis*RA were best described by a modified one-compartment, zero-order absorption model combined with an absorption lag time. Some patients had almost no absorption lag time followed by a very rapid absorption, whereas in other patients a prolonged lag time was observed, together with very slow absorption. Conversely, a very delayed but rapid rise in plasma concentrations was seen in some patients. Thus, a relatively complex model with an absorption lag time was required for all of the standard models. However, the simple first- and zero-order models did not fit particularly well when this approach was taken. Use of the modified zero-order model or the transit model provided a much better fit to the data.

The dosing regimen of 80 mg m^−2^ twice daily for 14 days was associated with significant inter-patient variation in 13-*cis*RA pharmacokinetics, comparable with that observed following ATRA administration for the treatment of APL (Lanvers *et al*, 2003). For 13-*cis*RA, pharmacokinetics may be influenced by a number of factors, including both the method of drug administration and the extent of metabolism.

Children diagnosed with high-risk neuroblastoma are commonly aged between 1 and 5 years, presenting a practical problem with regards to administering large numbers of 13-*cis*RA capsules. For example, an average child of 5 years with a surface area of 0.75 m^2^ would require a daily dose of 120 mg, administered as six large 20 mg capsules or 24 5 mg capsules. Younger children are therefore often unable to take 13-*cis*RA unless it is removed from the capsules before administration. In addition to the safety concerns that may arise from this practice, with regards to the handling of a potentially teratogenic substance, opening the capsule may impact on the actual dose of drug received. This may reflect loss of drug during handling in addition to the well-documented instability of 13-*cis*RA in the presence of light.

Of the 28 patients recruited to the current study, 17 (age 1.1–10.7 years) required the capsules to be opened and the contents mixed with food. Plasma concentrations of 13-*cis*RA in these patients were significantly lower than those observed in children who were able to swallow the capsules, although both groups exhibited a wide variation in plasma concentrations. No attempt was made to control food intake around the time of administration. A previous study has reported higher plasma concentrations when 13-*cis*RA was administered within 1 h before or after a standard meal, compared with the fasted state (Colburn *et al*, 1983). Application of the data presented here to guide dosing of 13-*cis*RA would also need to be adapted to reflect differences in the administration of 13-*cis*RA in different countries, for example punching a hole in the capsule so that it can be chewed before swallowing. The younger children, for whom the capsules were most likely to have been opened, also received the lowest absolute doses in milligrams.

Following oral administration, 13-*cis*RA may be subject to first-pass metabolism and subsequent plasma concentrations will depend on the rate and extent of metabolism to 4-oxo-13-*cis*RA, generally thought to represent a pathway of retinoid inactivation. Oxidative metabolism has previously been observed in studies with ATRA (Muindi *et al*, 1992; Rigas *et al*, 1993) and fenretinide (Villani *et al*, 2004), and auto-induction of oxidative metabolism has been associated with disease relapse, following chronic administration of ATRA in APL (Muindi *et al*, 1992). This can be avoided by the use of intermittent ATRA dosing schedules (Adamson *et al*, 1995), an approach that has also been recommended to minimise the side effects of ATRA in children (Lanvers *et al*, 2003). Our results with 13-*cis*RA show an accumulation of the 4-oxo-metabolite between days 1 and 14 of treatment in all patients, with metabolite concentrations higher than those of the parent drug by day 14 in approximately 70% of patients. There was no corresponding decrease in parent drug concentrations, which would have indicated enzyme induction. Thus, repeated dosing of 13*cis*RA is unlikely to be associated with cytochrome *P*450 (CYP) enzyme induction, as is seen with ATRA (Adamson *et al*, 1993).

With the limited size of the current study, it is difficult to obtain a clear indication of the impact of inter-patient pharmacokinetic variation and metabolism of 13-*cis*RA on clinical efficacy or toxicity. The degree of toxicity was similar to that reported in the Phase I study of 13-*cis*RA at the equivalent dose level (Villablanca *et al*, 1995). A statistically significant relationship was observed between the incidence of disease relapse and plasma metabolite concentrations, with an increased likelihood of relapse for patients with higher day 14 peak 4-oxo-13-*cis*RA levels. A corresponding decrease in parent drug was not detectable. It should be noted that older patients, who may have a higher risk of relapse, would receive higher doses of unopened capsules.

As lower doses of retinoic acid have been shown to be ineffective in the treatment of neuroblastoma (Matthay *et al*, 1999; Kohler *et al*, 2000), the extent of inter-patient pharmacokinetic variation following high-dose 13-*cis*RA therapy warrants further investigation. Individuals with plasma concentrations below those associated with antitumour activity may require an increased dose. One approach to overcome interindividual variation, and to ensure that plasma concentrations are not compromised by the method of administration, would be to monitor plasma concentrations on day 1 of treatment. Data in Figure 2 indicate that plasma concentrations do not vary substantially between days of treatment, thus dose adjustment based on the day 1 plasma concentrations is feasible.

It is notable that the plasma concentrations achieved in the current study are up to four-fold lower than those reported in a phase I study of the same formulation (Khan *et al*, 1996). The reasons for this difference are not easily identified. The age range, and thus the doses administered, in the phase I study population are not different to that studied here. It may be that the method of administration was different in the previous study, although the difference in AUC is equally marked in our patients who swallowed the capsules whole. There are differences in the assay for drug and metabolite in plasma, but our method was stringently validated and rigorous efforts were employed to minimise loss of drug due to photodegradation. Data on ATRA and the 4-oxo metabolite are comparable between the two studies, although little detail is given in the phase I report (Khan *et al*, 1996).

The conversion to 4-oxo-13-*cis*RA is the major route of 13-*cis*RA metabolism. Although there was no indication that this led to significantly lower parent retinoid levels during treatment, higher 13-*cis*RA levels may be achieved by limiting the extent of metabolism. Although oxidation of 13-*cis*RA may be mediated by a number of enzymes (Sonneveld *et al*, 1998; Nadin and Murray, 1999; Chen *et al*, 2000), specific inhibitors of retinoid metabolism, such as R116010, may be used to inhibit such oxidation (Armstrong *et al*, 2005) and thus may increase 13-*cis*RA plasma concentrations. These data also indicate that lower systemic 13-*cis*RA exposures are associated with the practice of opening the capsules and mixing the contents with food before administration. This finding highlights the importance of obtaining appropriate pharmaceutical formulations of medicines for children. Although a simple increase in 13-*cis*RA dose is likely to overcome this problem, it may be more appropriate to design a study incorporating the monitoring of plasma concentrations, with a view to standardisation of drug exposure and the development of more robust pharmacokinetic–pharmacodynamic relationships. The data presented here show that optimisation of dosing of 13-*cis*RA in high-risk neuroblastoma is possible, based on knowledge of the clinical pharmacology of this drug.

## Figures and Tables

**Table 1:** Patient characteristics

**Characteristic**	**No. of patients**	**%**
*Age (years)*		
<3	8	29
3–5	13	46
6–10	4	14
11–18	3	11
		
*Sex*		
Male	16	57
Female	12	43
		
*Weight (kg)*		
<10	1	3
10–20	21	75
20–30	3	11
>30	3	11
		
*Disease stage*		
3 (with *MYCN* amplification)	2	7
4 (age >1 year)	26	93
		
*MYCN amplification*		
Yes	15	54
No	13	46

**Table 2:** Comparison of population pharmacokinetic parameter estimates for various models; coefficient of variation (%) shown in paranthesis

**Model**	**OFV**	**CL/F**	**Ka**	**V/F**	**LAG/MTT**	**NN**	**D**
Zero-order absorption with lag time	50.1	10.53 (89.4)	—	111.1 (100.0)	27.39 (44.7)	—	57.40 (70.7)
First-order absorption with lag time	24.3	15.40 (63.8)	0.050 (236)	64.07 (102)	53.52 (11.7)	—	—
Modified zero-order absorption with lag time	13.8	15.87 (68.6)	0.068 (154)	84.77 (92.1)	40.04 (27.2)	—	11.8 (200.0)
Transit model	18.5	15.55 (63.9)	0.054 (207)	72.24 (93.5)	57.40 (52.2)	20.09 (36.2)	—

OFV, objective function value; CL/F, apparent clearance (l h^−1^); *K*_a_, absorption rate (1 min^−1^); *V*/*F*, apparent volume of distribution (l); LAG, absorption lag time (min); MTT, mean time to absorption transition (min); NN, number of transit compartments; *D*, absorption duration (min).

**Table 3:** Summary statistics for empirical Bayes pharmacokinetic parameter estimates on study day 1, analysed by course

**Parameter**	**Course**	**N**	**Mean**	**s.d.**	**Min**	**Median**	**Max**
CL/*F* (l h^−1^)	2	26	20.7	16.7	4.8	17.8	80.8
	4	12	12.5	6.7	3.4	11.1	24.7
	6	10	13.2	6.9	7.1	10.9	30.7
							
*V/F* (l)	2	26	121.1	128.7	21.6	75.0	490.2
	4	12	40.9	22.1	13.2	38.2	100.3
	6	10	68.8	57.2	30.1	41.4	194.4
							
AUC (*μ*mol h l^−1^)	2	26	13.2	9.0	2.1	10.5	37.3
	4	12	20.8	11.0	6.1	20.2	43.7
	6	10	17.2	8.1	5.4	15.8	31.3
							
CL/F (l h^−1^ m^−2^)	2	26	31.9	27.2	7.7	24.0	136.2
	4	12	17.7	11.4	5.9	13.4	41.0
	6	10	19.1	12.4	7.6	15.8	51.2
							
*V/F* (l m^−2^)	2	26	177.4	173.7	29.7	125.7	662.7
	4	12	58.8	39.7	21.7	51.3	168.3
	6	10	100.5	94.9	34.0	55.6	324.5
							
LAG (min)	2	26	41.2	10.6	23.7	39.0	57.7
	4	12	49.4	7.8	37.8	52.5	62.5
	6	10	43.7	7.5	31.9	44.1	54.5

CL/F, apparent clearance; LAG, absorption lag time; AUC, area under the curve, extrapolated to infinity; *N*, number of patients studied on each course of treatment. CL/*F* and *V/F* also given as values corrected for body surface area.

Course 2 refers to first available course. This was course 1 in 5 patients and course 3 in one patient.

**Table 4:** Summary statistics for 13-cisRA Cmax and 4-oxo-13-cisRA Cmax on study day 1 and 14, analysed by course

**Parameter**	**Course**	**N**	**Mean**	**s.d.**
*13-cisRA*				
Day 1 (*μ*M)	2	27	2.78	2.31
	4	13	5.43	2.23
	6	10	3.25	1.99
				
Day 14 (*μ*M)	2	27	2.83	1.44
	4	13	4.71	2.40
	6	10	2.52	1.85
				
*4-oxo-13-cisRA*				
Day 1 (*μ*M)	2	27	1.30	1.21
	4	13	1.84	0.85
	6	10	1.31	0.74
Day 14 (*μ*M)	2	27	4.67	3.17
	4	13	6.43	4.11
	6	10	3.49	1.98

Abbrevitions: RA=retinoic acid; s.d.=standard deviation.
